# Supplementary material for: Oxygen Vacancies in Bismuth Tantalum Oxide to Anchor Polysulfide and Accelerate the Sulfur Evolution Reaction in Lithium–Sulfur Batteries
Source: Nanomaterials (Basel). 2022 Oct 11;12(20):3551. doi: 10.3390/nano12203551 (PMC9607072; doi:10.3390/nano12203551)
Supplement: Supplementary file 1 [file nanomaterials-12-03551-s001.zip › nanomaterials-1947188-supplementary.pdf]

Supplementary Materials

Oxygen Vacancies in Bismuth Tantalum Oxide to Anchor Polysulfide and Accelerate the Sulfur Evolution Reaction in Lithium–Sulfur Batteries

Chong Wang [1,†], Jian-Hao Lu [2,†], An-Bang Wang [2], Hao Zhang [2], Wei-Kun Wang [2], Zhao-Qing Jin [2,*] and Li-Zhen Fan [1,*]

1 Beijing Advanced Innovation Center for Materials Genome Engineering, Institute of Advanced Materials and Technology, University of Science and Technology Beijing, Beijing 100083, China
2 Military Power Sources Research and Development Center, Research Institute of Chemical Defense, Beijing 100191, China
* Correspondence: jinzhaoqing1001@gmail.com (Z.-Q.J.); fanlizhen@ustb.edu.cn (L.-Z.F.)
† These authors contributed equally to this work.

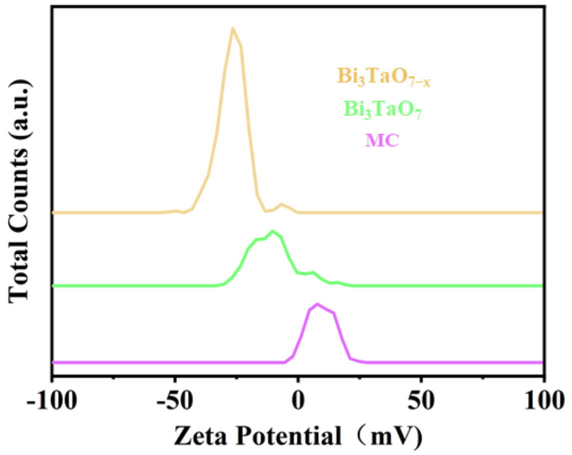

Figure S1. Zeta potentials of the dispersed three samples in water at pH value of 7.0.

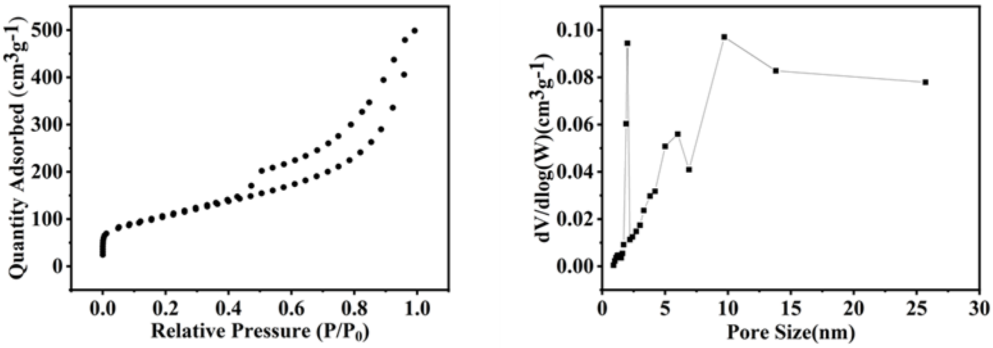

Figure S2. N_2 adsorption–desorption isotherm curves and the pore size distribution of Bi_3TaO_{7-x}.

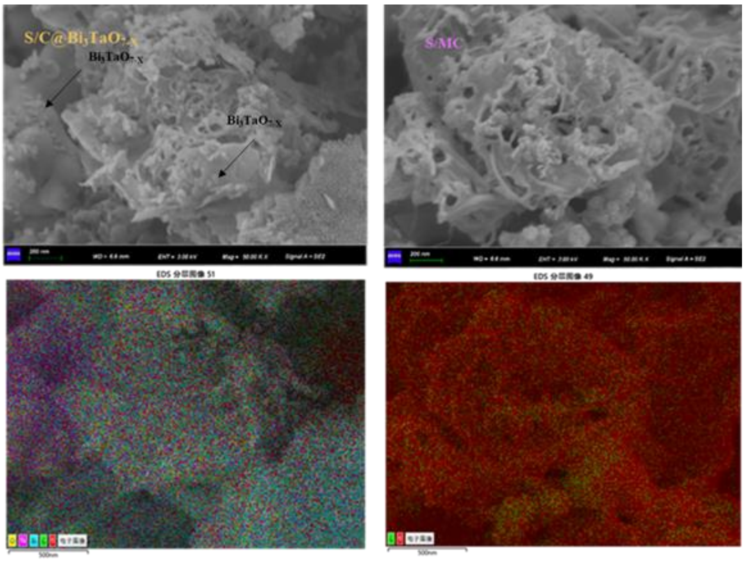

Figure S3. Characterization of SEM images and corresponding EDS elemental mappings of S/C and S/C@Bi_3TaO_{7-x}.

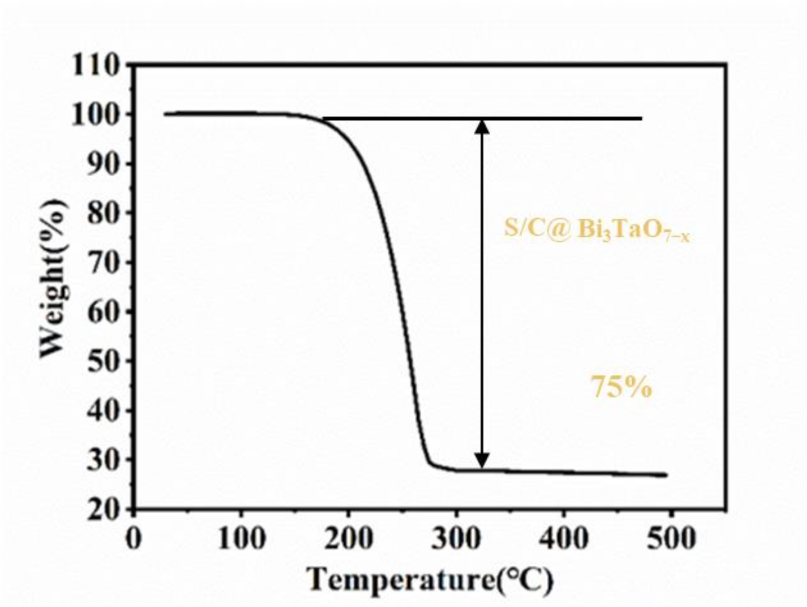

Figure S4. TGA curve of S/C@Bi_3TaO_{7-x}.

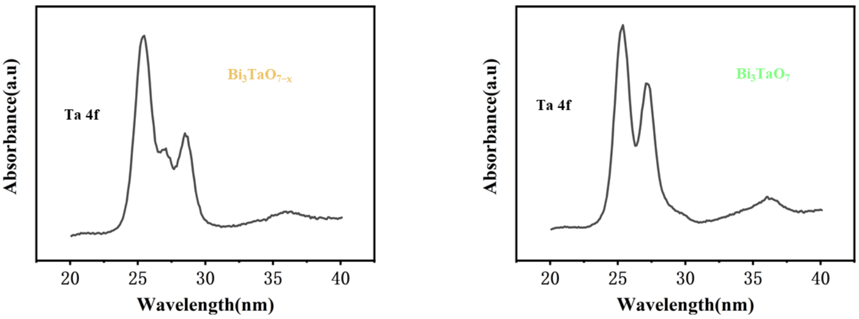

Figure S5. Ta 4f for Bi_3TaO_7 and Bi_3TaO_{7-x}.

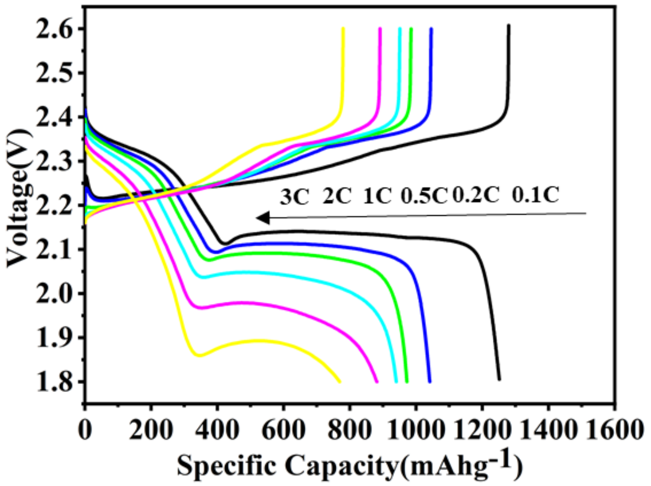

Figure S6. Multi-rate discharge-charge profiles of S/C@Bi_3TaO_{7-x}.

Table S1. The gravimetric energy is calculated based on all active and inactive components, including the packing film, current collectors, and separator.

Parameters	Unit	Value
**Sulfur loading**	%	80
**Carbon content**	%	10
**Binder content**	%	10
**Electrolyte/sulfur ratio**	μL mg^{-1}	3.3
**Number of double-sided coated cathode**		6
**Weight of tab**	g	0.28
**Weight of the sulfur**	g	2.82
**Weight of the packing plus pouch foil**	g	2.5
**Weight of the anode**	g	3.6
**Weight of the cathode**	g	4.70
**Weight of the separator**	g	0.64
**Weight of electrolyte**	g	9.30
**Total weight**	g	21.02
**Energy of cell**	Wh	6.3
**Energy Density**	Wh kg^{-1}	299

Table S2. Comparative table of Li-S pouch cell performance with recently reported works.

S loading (mg cm^{-2})	Energy density (W h kg^{-1})	E/S ratio (μL mg^{-1})	Areal capacity (mA h cm^{-2})	Capacity retention rate (%)	Cycle	References
4.0	206	6.0	4.8	~88	40	[1]
4.1	150	4.5	3.3	51	66	[2]
4.9	301	4.0	6.5	89	30	[3]
5.0	118	7.0	4.5	~77	50	[4]
6.0	317	4.0	7.2	74	80	[5]
6.1	301.4	3.0	7.1	81	23	[6]
6.5	299	2.5	5.9	83	10	[7]
13.6	303	2.4	11.0	78	30	[8]
**9.6**	**299.0**	**3.3**	**10.20**	**54**	**53**	**This work***

References

1. Zhao, M.; Chen, X.; Li, X. Y.; Li, B. Q.; Huang, J. Q. An organodiselenide comediator to facilitate sulfur redox kinetics in lithium–sulfur batteries. *Adv. Mater.* **2021**, *33*, 2007298.
2. He, J.; Bhargav, A.; Manthiram, A. High-Energy-Density, Long-Life Lithium–Sulfur Batteries with Practically Necessary Parameters Enabled by Low-Cost Fe–Ni Nanoalloy Catalysts. *ACS Nano* **2021**, *15*, 8583–8591.
3. Huang, Y.; Shaibani, M.; Gamot, T. D.; Wang, M.; Jovanović, P.; Cooray, D.; Majumder, M. A saccharide-based binder for efficient polysulfide regulations in Li-S batteries. *Nat. Commun.* **2021**, *12*, 1–15.
4. Luo, L.; Li, J.; Yaghoobnejad Asl, H.;Manthiram, A. In-situ assembled VS_4 as a polysulfide mediator for high-loading lithium–sulfur batteries. *ACS Energy Lett.* **2020**, *5*, 1177–1185.
5. Zhao, C.; Xu, G. L.; Yu, Z.; Zhang, L.; Hwang, I., Mo, Y. X.; Zhao, T. A high-energy and long-cycling lithium–sulfur pouch cell via a macroporous catalytic cathode with double-end binding sites. *Nat. Nanotechnol.* **2021**, *16*, 166–173.
6. Zhang, X. Q.; Jin, Q.; Nan, Y. L.; Hou, L. P.;Li, B. Q.; Chen, X.; Zhang, Q. Electrolyte Structure of Lithium Polysulfides with Anti-Reductive Solvent Shells for Practical Lithium–Sulfur Batteries, *Angew. Chem. Int. Ed.* **2021**, *60*, 15503–15509.
7. Qi, C.; Li, Z.; Wang, G.; Yuan, H.; Chen, C.; Jin, J.;Wen, Z. Microregion Welding Strategy Prevents the Formation of Inactive Sulfur Species for High-Performance Li-S Battery, *Adv. Energy Mater.* **2021**, *11*, 2102024.
8. Wang, X.; Yang, Y.; Lai, C.; Li, R.; Xu, H.; Tan, D. H.; Loh, K. P. Dense-Stacking Porous Conjugated Polymer as Reactive-Type Host for High-Performance Lithium Sulfur Batteries, *Angew. Chem. Int. Ed.* **2021**, *60*, 11359–11369.
